# p27^KIP1^ and PTEN cooperate in myeloproliferative neoplasm tumor suppression in mice

**DOI:** 10.1186/s40164-016-0047-0

**Published:** 2016-06-30

**Authors:** Jingchen Shao, Susann Li, Lars Palmqvist, Linda Fogelstrand, Stella Y. Wei, Kiran Busayavalasa, Kui Liu, Viktor M. Liu

**Affiliations:** 1 Department of Clinical Chemistry and Transfusion Medicine, Institute of Biomedicine, Sahlgrenska Academy, University of Gothenburg, Gothenburg, Sweden; 2 Laboratory of Clinical Chemistry, Sahlgrenska University Hospital, Gothenburg, Sweden; 3 Section for Haematology and Coagulation, Department of Medicine, Sahlgrenska University Hospital, Gothenburg, Sweden; 4 Department of Chemistry and Molecular Biology, University of Gothenburg, Gothenburg, Sweden

**Keywords:** PTEN, p27^KIP1^, Myeloproliferative neoplasms

## Abstract

PTEN acts as a phosphatase for PIP3 and negatively regulates the PI3K/AKT pathway, and p27^KIP1^ is a cyclin-dependent kinase inhibitor that regulates the G1 to S-phase transition by binding to and regulating the activity of cyclin-dependent kinases. Genetic alterations of *PTEN* or *CDKN1B* (p27^KIP1^) are common in hematological malignancies. To better understand how mutations in these two genes might cooperate in leukemogenesis, we inactivated both genes in the hematological compartment in mice. Here, we show that the combined inactivation of *Pten* and *Cdkn1b* results in a more severe myeloproliferative neoplasm phenotype associated with lower hemoglobin, enlarged spleen and liver, and shorter lifespan compared to inactivation of *Pten* alone. More severe anemia and increased myeloid infiltration and destruction of the spleen contributed to the earlier death of these mice, and elevated p-AKT, cyclin D1, and cyclin D3 might contribute to the development of this phenotype. In conclusion, PTEN and p27^KIP1^ cooperate in tumor suppression in the hematological compartment.

## Background

*PTEN* (phosphatase and tension homolog deleted on chromosome 10) is a tumor suppressor gene located on chromosome 10q23 and is one of the most commonly mutated or deleted genes in human cancers, including acute lymphoblastic leukemia, juvenile myelomonocytic leukemia, and non-Hodgkin’s lymphoma [1, 2]. PTEN acts as a phosphatase for phosphatidylinositol-3,4,5-trisphosphate (PIP3) and negatively regulates the phosphatidylinositol 3-kinase (PI3K)/AKT pathway [3]. The *CDKN1B* gene encodes p27^KIP1^, which belongs to the Cip/Kip family of cyclin-dependent kinase inhibitors. p27^KIP1^ is a key regulator of the G1 to S-phase transition by inhibiting cyclinD1/CDK4 and cyclinE/CDK2 complexes [4]. Deletions and other cytogenetic aberrations involving *CDKN1B* have been reported in a variety of leukemias [5–7]. In addition, *CDKN1B* expression can be a useful prognostic molecular marker for acute myeloid leukemia, where low *CDKN1B* expression is associated with high proliferation and, therefore, with a favorable response to chemotherapy [6]. Inactivation of the tumor-suppressor gene *PTEN* and lack of *CDKN1B* expression have been detected in some kinds of cancer, including most advanced prostate cancers and lymphomas [8, 9]. It has been shown that the combined loss of PTEN and p27^KIP1^ is associated with tumor cell proliferation and increased risk of recurrent disease in localized prostate cancer [10]. Loss of *PTEN* expression is more frequent in anaplastic large-cell lymphoma, which strongly correlates with the loss of *CDKN1B* expression [9].

Targeted disruption of the murine *Cdkn1b* gene causes a gene dose-dependent increase in animal size without other gross morphologic abnormalities [11], and deletion of *Pten* in the hematopoietic compartment in mice promotes excessive proliferation of leukemogenic stem cells resulting in the development of myeloproliferative neoplasm (MPN) followed by acute leukemia [12]. In mice, concomitant inactivation of *Pten* and *Cdkn1b* accelerates spontaneous neoplastic transformation of prostate cancer [8]. In order to better understand the relation and clinical relevance of these two genes in the pathogenesis of hematological malignancies, we used *Cre* recombinase to simultaneously inactivate *Pten* and *Cdkn1b* in the hematopoietic compartment.

## Results and discussion

**Fig. 1:**
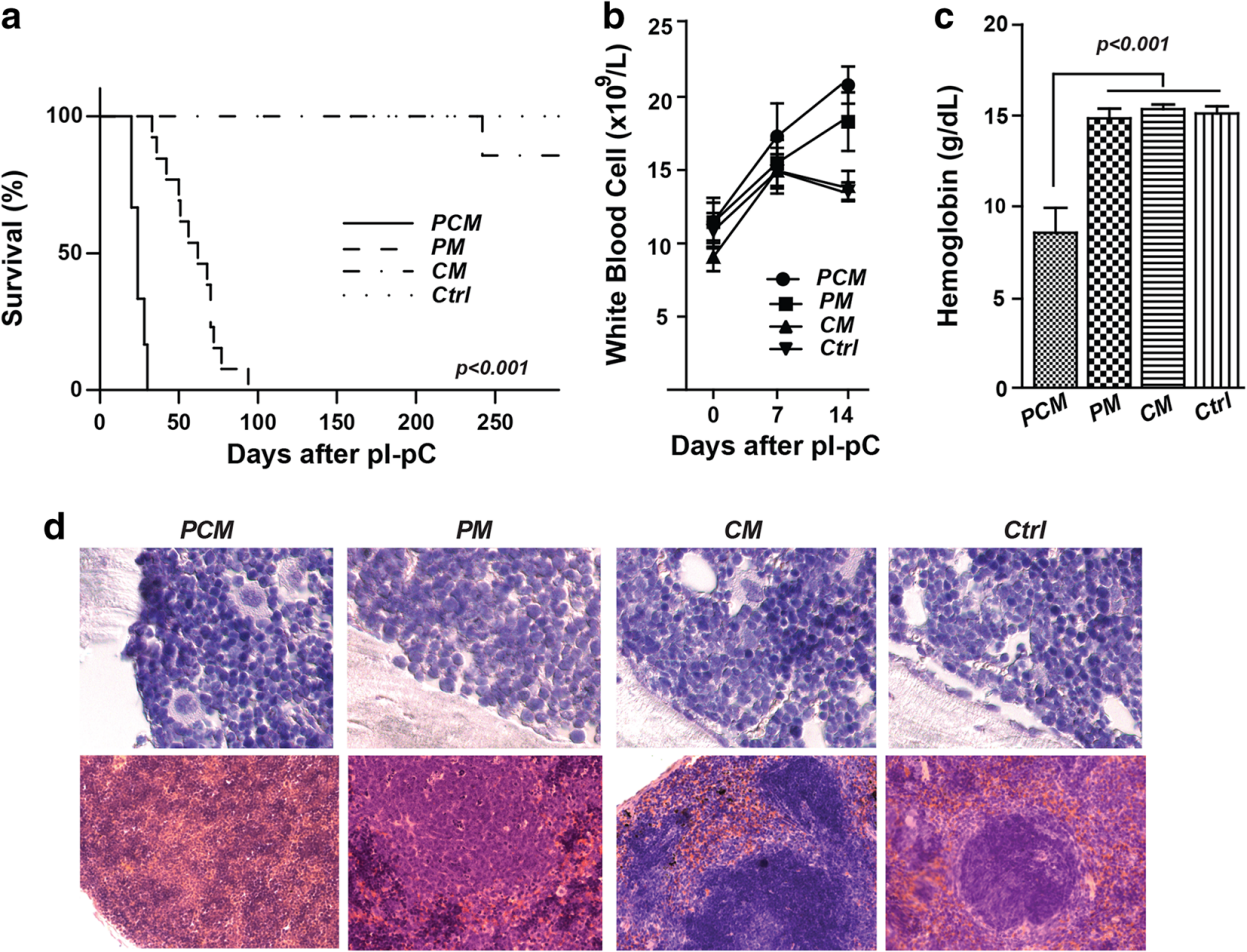
Survival, white blood cell counts, hemoglobin level, and histological analysis of all groups of mice. **a** Kaplan–Meier survival plots for *PCM* (n = 6), *PM* (n = 12), *CM* (n = 12) and *Ctrl* mice (n = 12). **b** White blood cell counts of *PCM*, *PM*, *CM* and *Ctrl* mice (n = 8 in each group). Blood was analyzed before and 1 and 2 weeks after pI–pC injections. **c** Blood hemoglobin concentrations of mice at 3 weeks after pI–pC injections. **d** Photographs of bone marrow (*top panels*) and spleen (*bottom panels*) sections (hematoxylin and eosin staining). Magnification was ×100/1.40 for bone marrow, ×20/0.50 for spleens

To determine the impact of combined deficiency of PTEN and p27^KIP1^ in the hematopoietic compartment, we injected pI–pC into *PCM*, *PM*, *CM* and *Ctrl* mice. Consistent with previous studies [13], all *PM* mice died from MPN by 98 days after pI–pC injections (median survival 62 days), whereas *CM* and *Ctrl* mice lived much longer and no MPN phenotype was observed in *CM* mice. However, the maximum survival of *PCM* mice was only 30 days (median 24 days; *p* < 0.001 versus *PM*; Fig. 1a). Two weeks after pI–pC injections, white blood cell counts were 20.8 × 10^9^ cells/L in *PCM* mice compared with mean counts of 18.3 × 10^9^, 13.9 × 10^9^ and 13.6 × 10^9^ cells/L for *PM*, *CM* and *Ctrl* mice, respectively (Fig. 1b). However, no morphological changes and no increase in the amounts of immature cells, including myeloblasts, could be detected in the blood and bone marrow in *PCM* mice compared with the other three groups (Fig. 1c, e). More severe anemia and more architectural disruption of the spleen were observed in *PCM* mice (Fig. 1d, e).

**Fig. 2:**
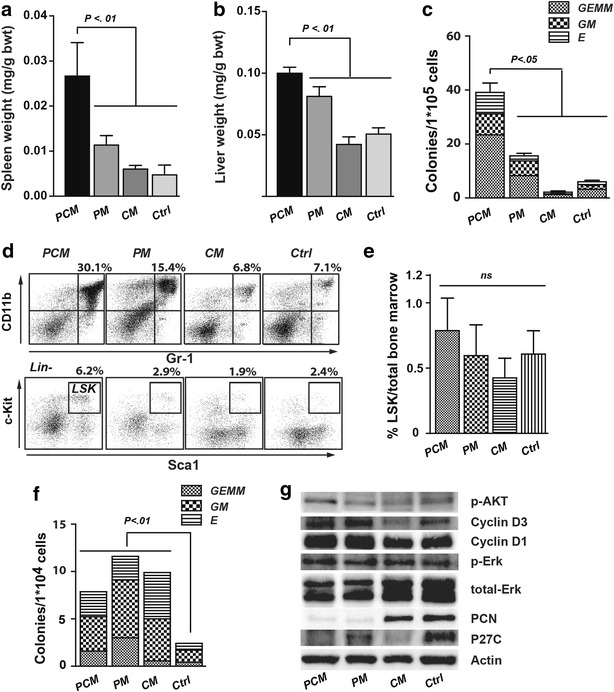
PCM mice exhibit massive hepatosplenomegaly, extramedullary hematopoiesis, and increased colony growth of splenocytes. **a** Spleen and **b** liver weight [relative to total body weight (bwt)] in *PCM* (n = 5), *PM* (n = 5), *CM* (n = 5), and *Ctrl* (n = 5) mice 3 weeks after pI–pC injections. **c** Colony-forming ability of splenocytes isolated from *PCM* (n = 3), *PM* (n = 3), *CM* (n = 3) and *Ctrl* (n = 3) mice around 3 weeks after pI–pC injections. *GEMM* colony-forming unit-granulocyte, erythroid, macrophage, megakaryocyte, *GM* colony-forming unit-granulocyte, macrophage, *E* burst-forming unit-erythroid. **d** Representative flow cytometry plots of splenocytes with antibodies recognizing CD11b, Gr-1, c-Kit, Sca1 and Lineage-negative. The mean percentage of double-positive splenocytes from *PCM* (n = 3), *PM* (n = 3), *CM* (n = 3), and *Ctrl* (n = 3) mice is indicated. **e** Percentage of LSK cells in bone marrow from *PCM* (n = 3), *PM* (n = 3), *CM* (n = 3), and *Ctrl* (n = 3) mice, as determined with flow cytometry. **f** Colony-forming ability of bone marrow from *PCM* (n = 3), *PM* (n = 3), *CM* (n = 3), and *Ctrl* (n = 3) mice. **g** Western blots of protein extracts from splenocytes of *PCM* (n = 2), *PM* (n = 2), *CM* (n = 2), and *Ctrl* (n = 2) mice at the 3rd week after pI–pC injection. Actin was used as the loading control

Spleen and liver weights in *PCM* mice increased by 2.3–5.6 and 1.2–2.4-fold, respectively, compared with *PM*, *CM* and *Ctrl* mice (Fig. 2a, b). Fluorescence-activated cell sorting analysis showed an increased proportion of CD11b+/Gr1+ and LSK [Lineage-negative (lin−), Sca-1+, c-Kit+] cells in the spleen of *PCM* mice compared to *PM*, *CM* and *Ctrl* mice (*p* < 0.05; Fig. 2c). Splenocytes from *PCM* mice produced more colonies compared with the other three groups (Fig. 2d). In bone marrow, there were no differences in the percentage of LSK cells (Fig. 2e). No increased colony formation in *PCM* mice was observed compared to *PM* mice when replated, and both groups had more colonies than the *Ctrl* mice when replated (Fig. 2f). Taken together, the phenotype in *PCM* mice is severe MPN rather than acute leukemia based on the criteria for classification of hematopoietic neoplasms in mice [14]. More severe anemia and increased myeloid infiltration and destruction of the spleen likely contributed to the earlier death of *PCM* mice compared with *PM* mice.

We performed Western blot analysis to determine the knock-out efficiency and the consequences of inactivating PTEN and p27^KIP1^ on downstream molecules. Deficiency of PTEN or p27^KIP1^ was observed in the respective knock-out mice (Fig. 2g). It has been shown that PTEN activity leads to the induction of p27^KIP1^, which in turn can negatively regulate the transition through the cell cycle [15]. However, the association between PTEN and p27^KIP1^ might be different in different kinds of tissues. A lack of convincing correlation between PTEN and p27^KIP1^ has been reported for ovarian carcinomas, indicating the possible existence of p27^KIP1^-independent pathways downstream of PTEN [16]. In our study, we found that expression of p27^KIP1^ was reduced in the splenocytes of *PM* mice. *PCM* mice had higher phosphorylated AKT compared to *PM*, *CM*, and *Ctrl* mice (Fig. 2g), and cyclin D1 and cyclin D3 expression levels were elevated in *PCM* and *PM* mice. However, the levels of phosphorylated ERK1/2 were similar in all groups of mice. Previous studies showed the synergistic activity of PI3K/mTOR and JAK2 signaling pathway in the myeloproliferative neoplasms [17, 18], therefore it will be interesting to study the JAK2 activity in PTEN and p27^KIP1^ knockout mice model.

In conclusion, our results show that PTEN deficiency can promote tumor progression by a decrease in p27^KIP1^ levels in the hematological compartment and that PTEN and p27^KIP1^ have a cooperative role in leukemia suppression. In addition, our results show that elevated phosphorylated AKT, cyclin D1, and cyclin D3 might play an important role in the progression of the severe MPN phenotype.

## Methods

### Animal procedures

Mice with conditional *Pten* ^fl/fl^ alleles (designated *P*) with a mixed genomic background of 129S4/SvJae and C57BL/6J were bred with *Cdkn1b* ^fl/fl^ mice (designated *C*) to generate *PC* mice. *PC* mice were bred with mice harboring the interferon (IFN)-inducible Mx1-*Cre* transgene (designated *M*) to generate *PCM* (*Pten* ^fl/fl^ *Cdkn1b* ^fl/fl^ Mx1-*Cre*), *PM* (*Pten* ^fl/fl^ Mx1-*Cre*), and *CM* (*Cdkn1b* ^fl/fl^ Mx1-*Cre*) mice. Mice without Mx1-Cre were used as healthy controls (designated *Ctrl*).

The mice were housed under controlled environmental conditions with free access to water and food. Illumination was on between 0600 and 1800 h. All mice were monitored daily. Groups of 4-week-old mice were injected with 400 µg polyinosinic-polycytidylic acid (pI–pC; Sigma, St Louis, MO). Blood was taken weekly and analyzed with a hematology analyzer KX-21 (Sysmex Europe, Norderstedt, Hamburg, Germany). Three weeks after injection, groups of mice were sacrificed and their tissues were harvested for further analysis. Mice were euthanized by cervical dislocation after carbon dioxide inhalation. In addition, groups of mice were kept for a survival study. If mice had ruffled fur and become listless or lost more than 10 % of their body weight, they were euthanized. All experimental protocols were approved by the regional ethical committee of the University of Gothenburg, Sweden.

### Genotyping

Genotyping was performed by PCR amplification of genomic DNA extracted from mouse tails. The *Pten* ^fl^ allele was detected with forward primer 5′-CAAGCACTCTGCGAACTGAG-3′ and reverse primer 5′-AAGTTTTTGAAGGCAAGATGC-3′, yielding a 328-bp fragment from the *Pten* ^fl^ allele and a 156-bp fragment from the *Pten* ^+^ allele. The *P27* ^fl^ allele was detected with forward primer 5′-TAGGGGAAATGGATAGTAGATGTTAGGACC-3′ and reverse primer 5′-GGTATAATATGGAAAGTGACTCTAATGGCC-3′, yielding a 400-bp fragment from the *P27* ^fl^ allele and a 370-bp fragment from the *P27* ^+^ allele. The Mx1-Cre transgene was detected with forward primer 5′-GCGGTCTGGCAGTAAAAACTATC-3′ (oIMR 1084) and reverse primer 5′-GTGAAACAGCATTGCTGTCACTT-3′ (oIMR 1085) to yield a 100 bp fragment.

### Fluorescence-activated cell sorting, colony assays, and histology

Splenocytes and bone marrow cells were incubated with antibodies against Gr1 (PE-Cy7/RB6-8C5), CD11b (V450/M1/70), c-kit (PE/2B8), Sca1 (PE-Cy7/D7), Lin- (FITC) and CD45 (V500/30-F11) and analyzed with FACS Diva software (BD Biosciences, San Jose, CA, USA). For colony assays, splenocytes (1 × 10^5^) and bone marrow cells (2 × 10^4^) harvested from experimental mice were seeded in duplicate wells in methylcellulose medium (MethoCult M3434; StemCell Technologies, Vancouver, BC, Canada). Six days later, the numbers of colonies were scored. For bone marrow cells, on the 7th day the cultured cells were washed, collected, and replated. Histology was performed as described [19, 20].

### Western blots

Tissue pieces (50–100 mg) were lysed in ice-cold buffer (50 mM Tris–HCl, 120 mM NaCl, 5 mM MgCl_2_, 1 % Triton X-100, 0.1 % sodium dodecyl sulfate, 1 % NP-40, 20 mM NaF, 1 mM phenylmethylsulfonyl fluoride, 2 mM orthovanadate, and the Complete Mini protease inhibitor cocktail). Lysates were homogenized, and centrifuged at 20,000*g* for 20 min, and equal amounts of total protein of the supernatant were size-fractionated on 10–15 % sodium dodecyl sulfate polyacrylamide gels. The proteins were transferred onto nitrocellulose membranes and incubated with antibodies against phosphorylated ERK1/2 (9106), total ERK (9102), phosphorylated AKT (9271), PTEN (9559), p27^KIP1^ (2552; Cell Signaling, Danvers, MA), Cyclin D1(sc-718), Cyclin D3 (sc-182), and Beta-actin (sc-47778; Santa Cruz Biotechnology, Inc., Dallas, TX). Protein bands were visualized with a horseradish peroxidase-conjugated secondary antibody (170-5046 and 170-5047; Bio-Rad Laboratories, Inc., Hercules, California) and the Enhanced Chemiluminescence Kit (Amersham, Little Chalfont, Buckinghamshire, United Kingdom). Band density was measured by Quantity One software (Bio-Rad Laboratories, Inc. USA).

### Statistical analyses

Data are plotted as the mean ± SEM. Differences in the concentrations and percentages of white blood cells, the colony-forming ability of hematopoietic cells, and the proliferation of cells in culture were determined with Student’s t test. Differences in mouse survival were assessed by the Mann–Whitney *U* test.

## Abbreviations

PTEN: phosphatase and tensin homolog
CDKN1B: cyclin-dependent kinase inhibitor 1B
PI3K: phosphatidylinositol-4,5-bisphosphate 3-kinase
AKT(PKB): protein kinase B
PIP3: phosphatidylinositol-3,4,5-trisphosphate
MPN: myeloproliferative neoplasm
pI–pC: polyinosinic–polycytidylic
PCM: Pten^fl/fl^ Cdkn1b^fl/fl^ Mx1-Cre
PM: Pten^fl/fl^ Mx1-Cre
CM: Cdkn1b^fl/fl^ Mx1-Cre
Ctrl: Control
LSK: lineage-negative (lin−), Sca-1+, c-Kit+
ERK: extracellular signal–regulated kinase
mTOR: mechanistic target of rapamycin
JAK2: Janus kinase 2
SEM: standard error of the mean
